# Lack of UGT polymorphism association with idasanutlin pharmacokinetics in solid tumor patients

**DOI:** 10.1007/s00280-018-3741-2

**Published:** 2018-12-03

**Authors:** W. Venus So, Tai-Hsien Ou Yang, Xing Yang, Jianguo Zhi

**Affiliations:** Roche Innovation Center New York, New York City, New York 10016 USA

**Keywords:** Idasanutlin, MDM2 antagonist, UGT, Polymorphism

## Abstract

**Purpose:**

Idasanutlin is a selective small-molecule MDM2 antagonist. It activates the tumor suppressor TP53 and is in phase 3 clinical trial for acute myeloid leukemia. Nonclinical studies have shown that glucuronidation is the major metabolizing mechanism for idasanutlin and UGT1A3 is the major metabolizing enzyme. There are reported examples of UGT polymorphisms associated with drug metabolism or response. Thus, the aim of this analysis is to investigate if UGT polymorphism is associated with idasanutlin pharmacokinetics.

**Method:**

Idasanutlin clearance was derived and normalized from two phase I studies. Its clearance level was compared between patients with different genotypes at 44 non-monomorphic UGT SNPs. Several single-locus and multi-locus association analysis, including haplotype association analysis and pairwise SNP interaction (epistasis) analyses were performed to investigate if there is any association between UGT genotypes and idasanutlin clearance.

**Results and conclusion:**

A total of 69 patients who have both idasanutlin pharmacokinetic data and UGT genotyping data were analyzed for association. The major clearance enzyme for idasanutlin, UGT1A3, has no association with idasanutlin clearance. Further single-locus and multi-locus association analyses also suggest that no significant UGT polymorphism association with idasanutlin clearance can be detected with the current datasets. However, the possibility of association with rare allele(s) of UGT family genes cannot be excluded due to the limited sample size of the current phase I studies.

## Introduction

Idasanutlin is a potent and selective small-molecule MDM2 (murine double minute 2) antagonist in phase 3 clinical trial for refractory/relapsed acute myeloid leukemia (AML). It disrupts interaction between the tumor suppressor protein 53 (TP53) and its negative regulator, MDM2, hence restoring TP53’s function in cell cycle control. Treatment of cancer cells expressing functional TP53 with small molecule MDM2 antagonists resulted in the concurrent transcriptional activation of TP53 downstream genes, cell cycle arrest and apoptosis [1].

Pharmacokinetic properties of idasanutlin have been well characterized in phase I studies [2–4]. It has low clearance, 1-day half-life and dose linearity [3, 4]. Nonclinical metabolic profiling studies have also suggested that glucuronidation is the major metabolic clearance reaction for idasanutlin (unpublished).

UGT (UDP glucuronosyltransferase) is a family of metabolizing enzymes that catalyzes glucuronidation. They play a key role in removing exogenous chemicals such as drugs and toxins as well as endogenous substances such as bilirubin. There are multiple reported examples of known UGT polymorphisms that are associated with drug metabolism [5–8] and drug response [9, 10]. Thus, we investigate if UGT polymorphism has an impact on the metabolism of idasanutlin observed from our MDM2 clinical studies.

## Methods

### Patients

Two phase I studies [2, 4] with pharmacokinetic data from solid tumor patients receiving idasanutlin in different formulations and dose levels, and UGT genotyping data were analyzed. A total of 69 patients were included.

### UGT genotyping

UGT genotyping data were analyzed using DMET chip (Drug Metabolizing Enzymes and Transporters) at Covance Genomics Lab, Redmond, WA 98052, USA. The chip measures SNP (Single Nucleotide Polymorphism) data for over 230 genes known to encode metabolizing enzymes and transporters, including UGT.

### Pharmacokinetic data

Idasanutlin pharmacokinetic data following single-dose administration were measured using plasma samples collected from patients and were reported [2, 4].

### Data analysis

#### Pharmacokinetic data analysis

Idasanutlin clearance (dose/AUC_inf_) from different formulations [MBP (Microprecipitated Bulk Powder) and SDP (Spray Dry Powder)] was normalized using the established dosage conversion: MBP/2.2 = SDP. Normalized clearance data were log transformed.

#### SNP–SNP association

Pairwise SNP–SNP associations were analyzed using the *r*^2^ measure implemented in the LDheatmap R package [11].

#### Single-locus association analysis

Normalized and log-transformed idasanutlin clearance data from all patients were pooled and analyzed by Welch’s ANOVA to detect statistical significance between genotypes and normalized clearance. Single-locus model using Fast score test for genetic association (qtscore function in GenABEL package) was also used to confirm results [12].

#### Multi-locus association analysis

Multi-locus association with normalized clearance was tested through a stepwise linear regression model based on Multi-Locus Mixed Model (MLMM) [13]. Missing values were imputed by K-nearest neighbor imputation with *k* = 10. Haplotype association analysis was carried out using haplo.score and seqhap functions in haplo.stats R package [14, 15]. The normalized clearance (log transformed) was binarized at its global median and the median of each patient group for seqhap haplotype association analysis.

Potential interaction between pairs of SNPs (epistasis) on normalized clearance was tested using interactionPval function in SNPassoc R package [16].

#### Software and computational approach

R version 3.4.0 was used for all the data analysis.

## Results and discussion

### Patient groups

This analysis includes data from two phase I studies in which patients received idasanutlin in different formulations and dose levels. Study 1 (NCT01901172) has three parts of clinical pharmacology study, with a total of 61 solid tumor patients [2]. Treatment A part in study 2 (NCT02828930) has 8 solid tumor patients with equivalent clinical pharmacology data [4].

### Pharmacokinetic data overview

Clearance of idasanutlin in different formulations was normalized and its distribution was normally distributed (Shapiro test) after log transformation. The clearance profiles from part 1 and 3 in study 1 and from study 2 were comparable, whereas the clearance from part 2 in study 1 was slightly higher, possibly due to inter-patient variability.

### UGT polymorphism

There are 115 SNPs in 17 UGT family enzymes on the DMET chip. Most of the SNPs do not show polymorphism among the 69 patients in the dataset. Only 44 of the 115 SNPs have more than one patient having a different genotype (non-monomorphic) and have reference SNP ids (rs#, [17]). Polymorphism of these 44 SNPs was analyzed for association with idasanutlin pharmacokinetic data.

### UGT1A3 polymorphism showed no association with idasanutlin clearance

**Fig. 1:**
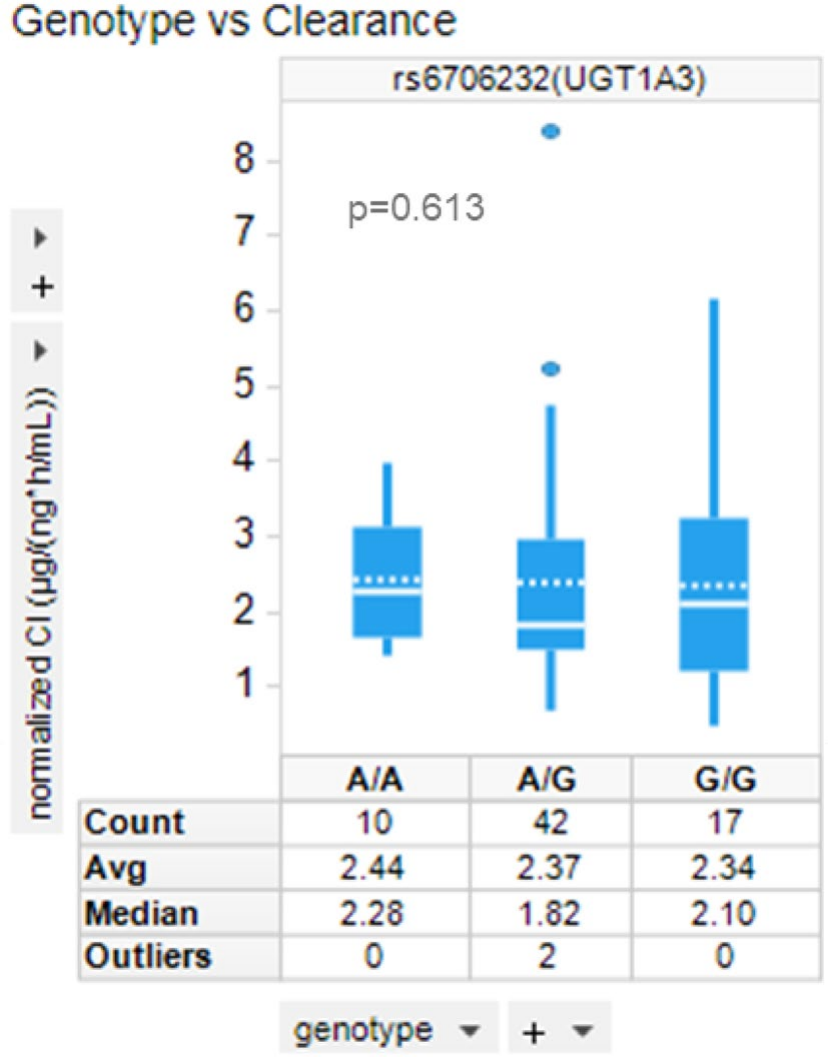
Idasanutlin clearance distribution in patients with different genotypes at a UGT1A3 SNP. Distribution of normalized clearance of idasanutlin among patients with the different genotypes at rs6706232 (one of the three measured SNPs in UGT1A3 that has polymorphism among patients in this dataset)

Previous in-house phenotyping assays with recombinant UGTs in human liver and intestinal microsomes and with selective UGT inhibitors have shown that UGT1A3 was the most active metabolizing enzyme for idasanutlin (data not shown). Therefore, we first analyzed the polymorphisms in UGT1A3. Three out of the six measured UGT1A3 SNPs have more than one patient showing polymorphism in the dataset: rs3821242, rs6706232, rs7574296. They are in strong linkage disequilibrium (*r*^2^ > 0.96). Therefore, the allele frequencies and idasanutlin clearance distribution in the different genotypes are very similar among the three SNPs. The box plot in Fig. 1 shows that there is no significant difference in the level of normalized clearance in patients with the three different genotypes at these SNPs, using one of them as an example (rs6706232). Therefore, there is no UGT1A3 polymorphism association with idasanutlin clearance (Welch’s ANOVA *p* value = 0.613).

### None of the UGT polymorphisms has association with idasanutlin pharmacokinetics

We then performed multiple single-locus and multi-locus association analyses to detect if there is any UGT polymorphism that is associated with idasanutlin clearance.

None of the UGT polymorphisms showed significant association with normalized clearance (Welch’s ANOVA *p* value > 0.05). Welch’s ANOVA was used because the number of patients is often different between the different genotypes, resulting in unequal variances for each SNP. Welch’s ANOVA does not assume equal variance among genotypes. The not-significant association was confirmed with a single-locus model using Fast score test for genetic association (qtscore function [12]).

To detect any association from multiple loci, each has very small effect on idasanutlin pharmacokinetics, we performed multi-locus association analysis. The stepwise linear regression model based on Multi-Locus Mixed Model (MLMM) [13] adjusted for kinship did not find any SNP significantly associated with normalized clearance.

Haplotype association analysis is also known to improve detection of genetically associated genes in some cases [18]. We tested haplotype association using three different ways of defining the haplo-block: (1) all non-singular SNPs in each gene were combined as a haplo-block; (2) all non-singular SNPs in each chromosome were combined as a haplo-block; (3) we used the sequential scan seqhap function to enlarge the locus region for haplotype association. Normalized clearance of idasanutlin was evaluated for association with the resulting haplotypes from the above three approaches. None of the UGT genes has a significant association with clearance based on global score statistics and permutation (*p* value > 0.05, data not shown).

**Fig. 2:**
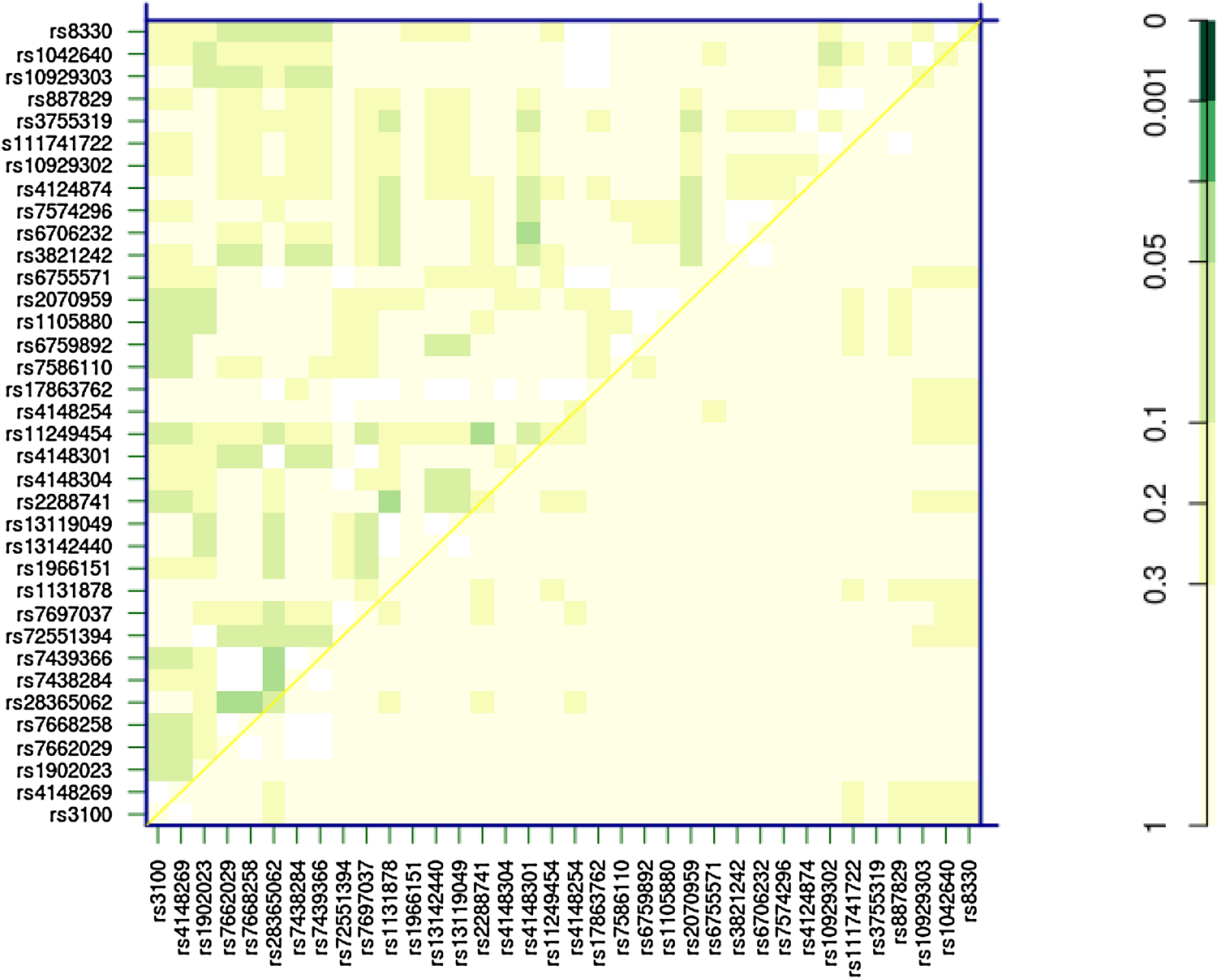
Potential pairwise SNP interaction on idasanutlin clearance. The heatmap shows *p* value (not adjusted for multiple testing) of pairwise interactions. The upper triangle, the lower triangle, and the diagonal present the *p* values of the SNP–SNP interactions, the differences of the additive models and the best single-SNP models, and the single-SNP effects, respectively. Darker color indicates higher significance (lower *p* value). Only the non-monomorphic SNPs with ≥ 80% non-missing genotypes are presented in the heatmap

We further evaluated potential interaction between pairs of SNPs (epistasis) on normalized clearance. Seven of the 44 non-singular SNPs showed some mildly significant epistasis (*p* value < 0.05, Fig. 2). However, after adjusted for multiple testing (by False Discovery Rate), none of them passes the < 0.05 significant *p* value cut-off.

We therefore conclude that with the current phase I study data, no significant UGT polymorphism association with idasanutlin clearance can be detected. This does not exclude the possibility that there might be some very rare alleles in UGT family gene(s) that associate with idasanutlin pharmacokinetics, due to the limited sample size in phase I studies. Samples from phase III studies can be collected to continue the investigation.
